# A comparison of reproductive isolation between two closely related oak species in zones of recent and ancient secondary contact

**DOI:** 10.1186/s12862-019-1399-y

**Published:** 2019-03-06

**Authors:** Wan-Jin Liao, Bi-Ru Zhu, Yue-Fei Li, Xiao-Meng Li, Yan-Fei Zeng, Da-Yong Zhang

**Affiliations:** 1 State Key Laboratory of Earth Surface Processes and Resource Ecology and MOE Key Laboratory for Biodiversity Science and Ecological Engineering, College of Life Sciences, Beijing Normal University, Beijing, 100875 China; 2 Key Laboratory of Tree Breeding and Cultivation of the State Forestry Administration, Research Institute of Forestry, Chinese Academy of Forestry, Beijing, 100091 China

**Keywords:** Ancient contact zone, Hybridization, Postzygotic, *Quercus*, Recent secondary contact, Reproductive isolation, Temporal differentiation

## Abstract

**Background:**

Much of the debate over the evolutionary consequences of hybridization on genetic divergence and speciation results from the breakdown or reinforcement of reproductive barriers in secondary hybrid zones. Among hybrid populations established for different lengths of time following secondary contact, stronger reproductive barriers are generally expected to occur in zones with longer contact. However, in plants no detailed investigation of recent and ancient zones of secondary contact has been conducted despite the importance of such a comparative study. Here, we compare pre- and postzygotic reproductive barriers between two closely related oak species, *Quercus mongolica* and *Q. liaotungensis*, in such a situation.

**Results:**

The recorded flowering times of both species overlapped in both contact zones. The fruit set at 10 and 30 days after interspecific hand pollination was not significantly lower than that after intraspecific pollination whenever *Q. mongolica* or *Q. liaotungensis* comprised the maternal parents in both populations. These results indicated that neither prezygotic phenological barriers nor interspecific incompatibility could have resulted in the reproductive isolation between the two species in both hybrid zones. However, the proportion of hybrid seeds produced by both species in the ancient zone was significantly lower than that recorded in the recent zone of secondary contact. In addition, the proportion of hybrid seeds simulated to form, assuming both random mating and an absence of postpollination barriers, was significantly higher than that detected in the ancient contact zone but not in the recent contact zone. These results suggest stronger early-acting postzygotic isolation between the two oak species in the ancient relative to the recent contact zone.

**Conclusions:**

Our comparative study demonstrated that postzygotic barriers during seed maturity were the main contributing factor to total reproductive isolation, particularly in the ancient contact zone, which aided species delimitation. In the recently formed secondary contact zone, pre- and postzygotic barriers were not well developed, and a high frequency of natural hybridization was evident. To our knowledge this study provides the first comparison of reproductive isolation between the ancient and recent secondary contact zones in plants and helps to clarify the evolutionary consequences of hybridization in a temporal context.

**Electronic supplementary material:**

The online version of this article (10.1186/s12862-019-1399-y) contains supplementary material, which is available to authorized users.

## Background

Hybridization occurs in approximately one-quarter of plants [1] and plays an important role in plant speciation and gene flow among genetically distinct populations and taxa [1–5]. Theoretical and empirical studies suggest that hybridization may accelerate genetic divergence and speciation by creating genetic novelty [2–4, 6–9] or may inhibit differentiation because of the breakdown of reproductive barriers [10, 11]. Comparisons of reproductive isolations between hybrid populations in ancient and recent secondary contact zones may help to clarify the evolutionary consequences of hybridization in a temporal context [2, 12]. When incompletely isolated populations or taxa are in geographic contact again, reproductive barriers to gene flow may break down, leading to a reduction of genetic differentiation and even the challenge of species delimitation [13, 14]. The opposite outcome is that any reproductive barriers might be strengthened under selection against unfit hybrids [15, 16]. Moreover, these consequences may vary among hybrid populations established for different lengths of time following secondary contact. Unfortunately, whether barriers to gene flow are strengthened or broken down in secondary contact zones remains uncertain, although theoretical and empirical evidence suggests that the breakdown of reproductive barriers is more likely [2]. Significantly, no study to our knowledge has compared barriers to reproductive isolation in ancient and recently formed plant hybrid zones.

Reproductive isolation is of central interest for understanding speciation and diversification [17–20]. An increasing number of case studies suggest that a range of pre- and postzygotic isolating barriers contribute positively to reproductive isolation between closely related taxa [12], and researchers have attempted to quantify the contribution of each barrier to reproductive isolation [20–22]. The general expectation is that prezygotic isolation is more likely to occur and contributes more to total reproductive isolation [17, 20, 23]; however, changes in species ranges may significantly erode prezygotic isolation and increase the frequency of hybridization [2, 23]. On the other hand, postzygotic isolation is thought to be well characterized at the genetic level and prevalent [18, 20, 24, 25]. If postzygotic isolation is more easily formed and strengthened as a function of genetic divergence [20, 24, 26, 27], it is reasonable to expect that postzygotic isolation would evolve more rapidly [25] and would therefore be stronger in ancient than in recent contact zones. So far, only for a few taxa that form hybrid zones have detailed analyses been conducted on the nature of pre- and postzygotic reproductive barriers, while no investigations have been conducted for most hybrid zones [12]. Furthermore, few studies have focused on the differential performance of reproductive isolation among regions in which closely related species exhibited different hybridization rates or co-occurrence history. Only one study compared the isolating barriers in *Ipomopsis* zones with varied hybridization rates and found that the prezygotic pollinator choice of flowers promoted different rates of hybridization [28]. Therefore, comparative studies on reproductive isolation between ancient and recently formed plant hybrid zones are crucial to understand the evolutionary consequences of hybridization in a temporal context.

Oak species are widely distributed in the Northern Hemisphere from cool temperate to tropical latitudes and show a high frequency of interspecific hybridization [12, 29–34]. Our previous studies have revealed an ancient secondary contact zone in Northeast China and a recent secondary contact zone in North China between two closely related oak species, *Quercus mongolica* and *Q. liaotungensis*, which provided a valuable study system for comparing the evolutionary consequences of hybridization in a temporal context [34]. We proposed that selection against hybrids may have had sufficient time to reinforce the reproductive barriers in the ancient but not in the recent secondary contact zone [34].

Here, we evaluated the evolutionary consequences of hybridization in a temporal context by comparing the pre- and postzygotic reproductive isolations between *Q. mongolica* and *Q. liaotungensis* in zones of recent and ancient secondary contact. We investigated ecological isolation by recording the flowering phenology and interspecific compatibility by following the fruit set after inter- and intraspecific hand pollination. In addition, we scored genotypes of mature seeds at seven microsatellite loci and calculated the proportion of hybrid seeds under natural and simulated pollination conditions to evaluate the postzygotic predispersal isolation. We systematically sampled individual trees throughout the populations, identified the species status of each sample, and calculated the proportion of hybrids to evaluate whether postdispersal isolation decreased the proportion of hybrids during their growth. The specific questions we addressed were: 1) Do pre- or postzygotic barriers contribute more to total reproductive isolation between *Q. mongolica* and *Q. liaotungensis*? and 2) Are reproductive barriers weaker in the recent secondary contact zone?

## Results

### Flowering phenology

The flowering phenology overlapped between the two *Quercus* species in both the NA (ancient) and Dlw (recent) populations, and every investigated tree had the potential to hybridize with the other species according to their flowering phenology in both the NA and Dlw populations. In 2012 in population NA, the four *Q. liaotungensis* trees began dispersing pollen on 17th May and ceased on 23rd May, whereas the three *Q. mongolica* trees began dispersing pollen on 18th May and ceased on 23rd May. In 2013 in population Dlw, the 16 *Q. liaotungensis* trees began dispersing pollen on 12th May and ceased on 18th May, whereas the seven *Q. mongolica* trees began dispersing pollen on 13th May and ceased on 21st May.

### Fruit set under intra- and interspecific pollination

**Fig. 1:**
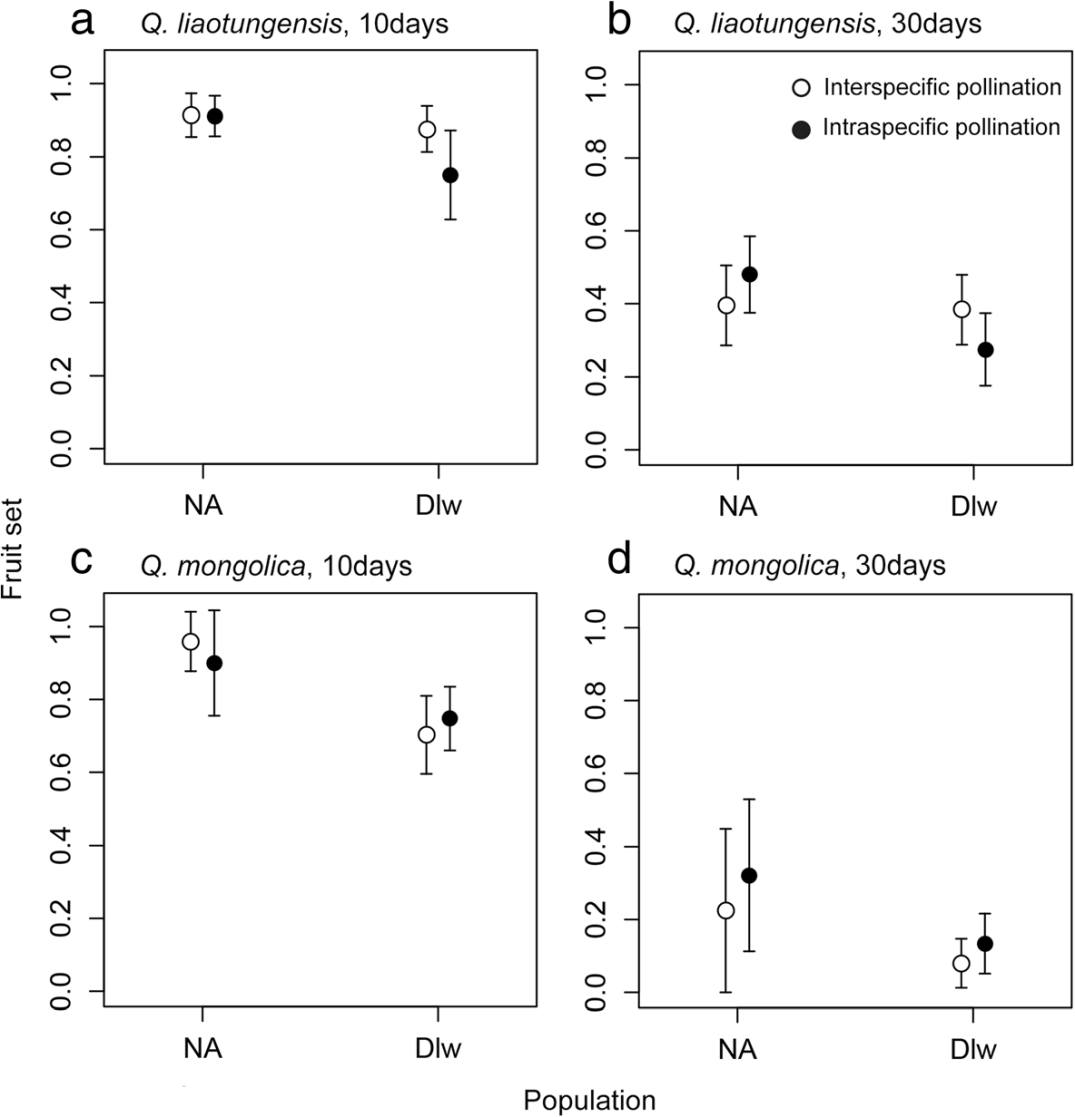
Fruit set (mean ± SE) at 10 and 30 days after inter- and intraspecific hand pollination in the ancient and recent secondary contact zone between *Quercus liaotungensis* and *Q. mongolica*. The open and solid circles represent fruit set after inter- and intraspecific pollination, respectively. Figures **a** and **b** indicate the fruit set at 10 and 30 days after pollination when *Q. liaotungensis* comprised the maternal plants, respectively, and the Figures **c** and **d** indicate the fruit set at 10 and 30 days after pollination when *Q. mongolica* comprised the maternal plants, respectively

Both *Q. liaotungensis* and *Q. mongolica* were compatible when hand pollinated with interspecific pollen (Fig. 1). The full model suggested that intraspecific pollination had negative effect on the fruit set, while population had no significant effect on the fruit set (Additional file 1: Table S1). However, the interaction between population and pollination treatment indicated that intraspecific pollination significantly increased the fruit set in population NA but not in population Dlw (*P* = 0.001, Additional file 1: Table S1).

The maternal species had a significant effect on the fruit set (Additional file 1: Table S1) and the interaction between maternal species and pollination treatment indicated that the fruit set following intraspecific pollination were significantly more than that following interspecific pollination when *Q. mongolica* comprised the maternal plants (*P* < 0.001). When *Q. liaotungensis* comprised the maternal plants, the interaction between population and pollination treatment suggested different scenarios in the two populations (Additional file 1: Table S1). In population NA, the fruit set following intraspecific pollination was higher than that following interspecific pollination (*P* = 0.002); while in population Dlw, the fruit set following intraspecific pollination was lower than that following interspecific pollination (*P* < 0.001, Fig. 1).

### Hybridization rate under open and simulated pollination conditions

**Fig. 2:**
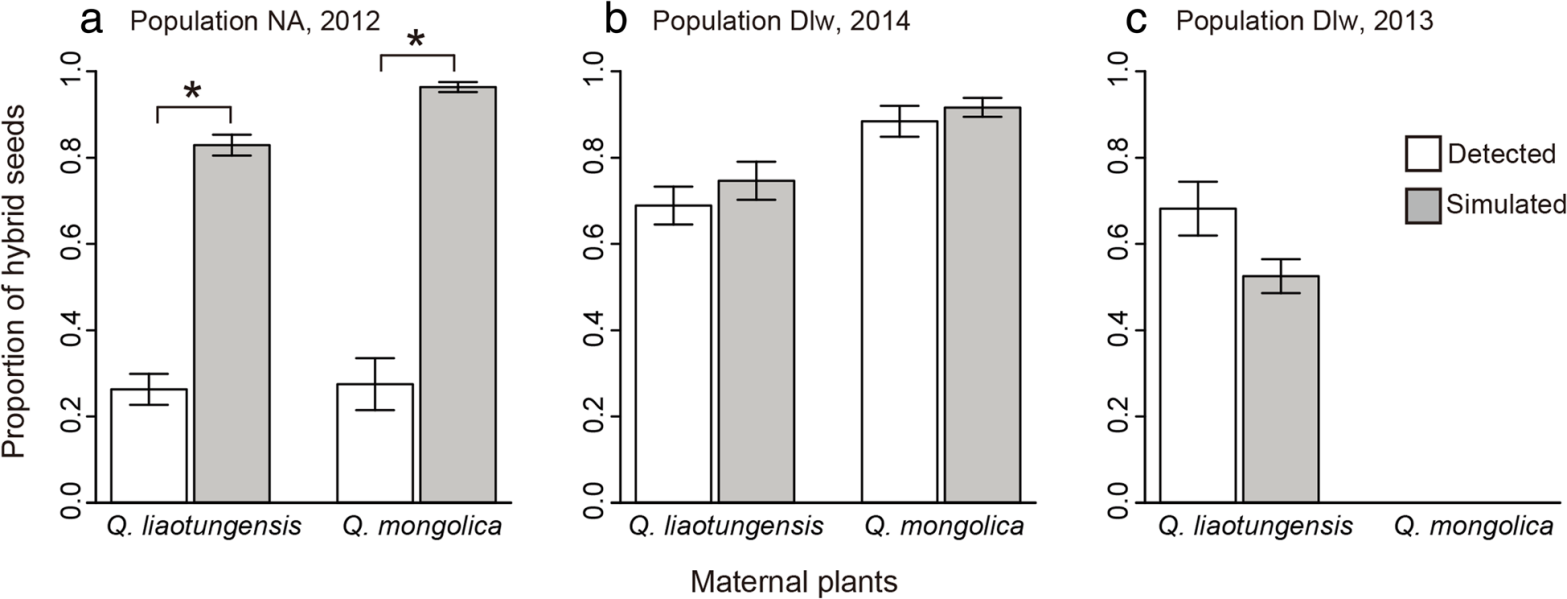
Proportion of hybrid seeds (mean ± SE) under natural and simulated conditions in populations NA and Dlw. The investigation was conducted in population NA in 2012 (**a**) and in population Dlw in both 2013 (**c**) and 2014 (**b**). The * indicates significant difference in the proportion of hybrid seeds under natural and simulated conditions

Both *Q. liaotungensis* and *Q. mongolica* trees produced hybrid seeds under open pollination in both populations (Fig. 2), however the proportion of hybrid seeds significantly differed between populations and between maternal species (Additional file 2: Table S2). The proportion of hybrid seeds in population NA (*Q. liaotungensis*: 0.263 ± 0.036; *Q. mongolica*: 0.275 ± 0.060) was much less than that (*Q. liaotungensis* in 2013 and 2014: 0.682 ± 0.062 and 0.689 ± 0.044; *Q. mongolica*: 0.885 ± 0.036) in population Dlw (*P* < 0.001). The *Q. mongolica* trees tended to have higher proportion of hybrid seeds (*P* < 0.001). The interaction between population and maternal species indicated that in population NA the proportion of hybrid seeds decreased more when *Q. mongolica* comprised the maternal species (*P* = 0.001).

Based on the simulated seed multilocus genotypes and Bayesian analyses, the simulated proportion of hybrid seeds without postpollination barriers in 2013 in population Dlw was 0.525 ± 0.039 for *Q. liaotungensis* and in 2014 was 0.747 ± 0.044 for *Q. liaotungensis* and 0.917 ± 0.022 for *Q. mongolica*. The simulated proportion of hybrid seeds was not significantly different from the detected proportion based on the molecular data set (Fig. 2). However, the simulated proportion of hybrid seeds was 0.829 ± 0.024 for *Q. liaotungensis* and 0.963 ± 0.012 for *Q. mongolica* in population NA, both of which were significantly higher than the actual proportion of hybrid seeds for both species (*Q. liaotungensis*: t = 19.970, *P* < 0.001; *Q. mongolica*: t = 10.624, *P* < 0.001; Fig. 2).

### Hybrid growth costs

**Fig. 3:**
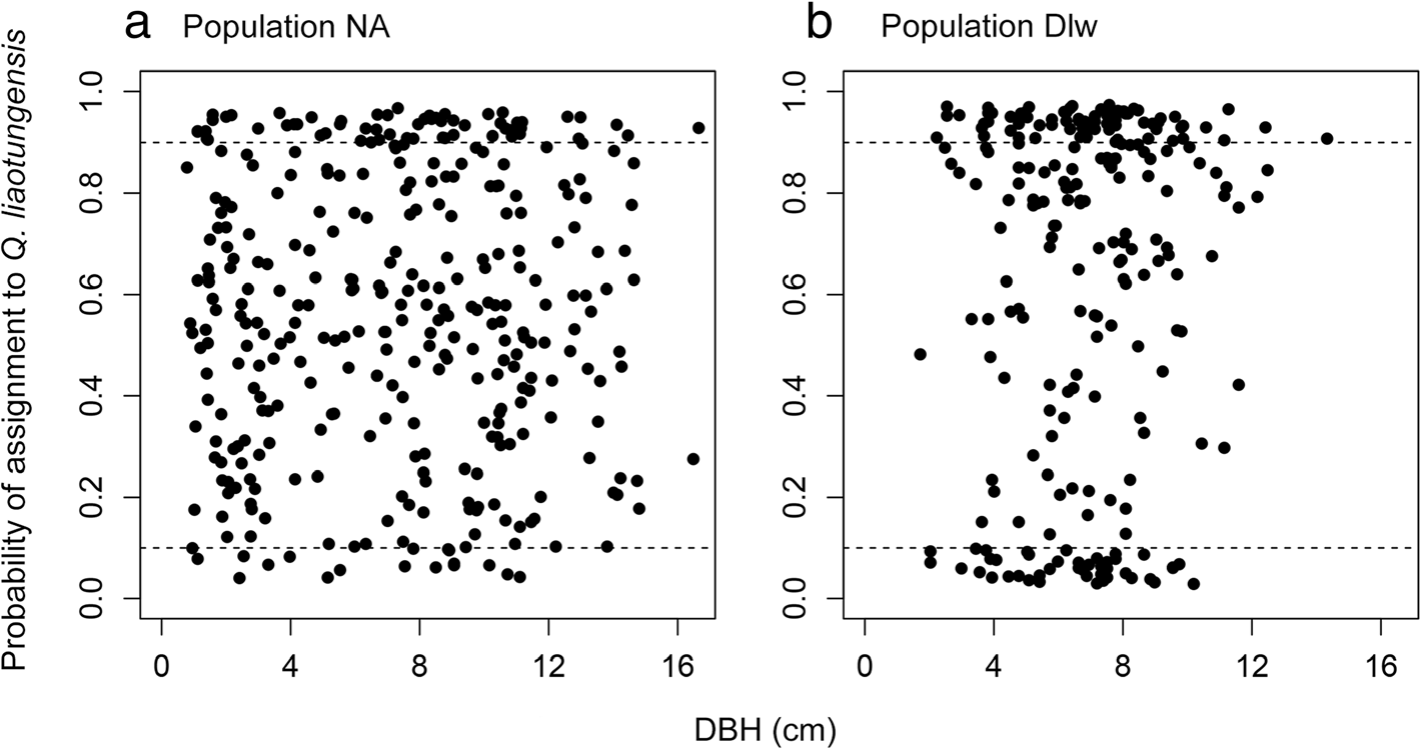
Relationship between tree diameter at breast height (DBH) and the probability of a tree being assigned to *Q. liaotungensis* in populations NA (**a**) and Dlw (**b**). The dashed lines indicate the cut-off value of 0.9. Scatter points above the 0.9 dashed line represent *Q. liaotungensis* trees, scatter points beneath the 0.1 dashed line represent *Q. mongolica* trees, and scatter points between the two lines represent the hybrid trees

Based on the assignment analyses, the individuals sampled from population NA included 80 *Q. liaotungensis* trees, 44 *Q. mongolica* trees, and 119 hybrids. Similarly, the individuals sampled from population Dlw included 59 *Q. liaotungensis* trees, 17 *Q. mongolica* trees, and 265 hybrids. The proportion of hybrid trees in population NA was 0.490, which was significantly lower than that in population Dlw (0.777) (*Χ*^*2*^ = 50.78, df = 1, *P* < 0.001). In population NA, the diameter at breast height (DBH) distribution of hybrids was not significantly different from that of individuals of both species (hybrid DBH: 7.00 ± 0.21 cm; both species DBH: 6.75 ± 0.20 cm; D = 0.095, *P* = 0.641). A similar DBH distribution was found in population Dlw (hybrid DBH: 7.35 ± 0.25 cm; both species DBH: 7.32 ± 0.42 cm; D = 0.106, *P* = 0.521). In both populations, logistic regression results revealed that the probability of randomly sampling a hybrid tree from the population did not decrease with increasing DBH (NA: z = 0.856, *P* = 0.392; Dlw: z = 0.066, *P* = 0.948). Our results suggest that hybrids did not suffer increased growth costs in either the NA or Dlw population (Fig. 3).

## Discussion

Our comparative study of reproductive isolation between the ancient and recent secondary contact zones of two closely related oak species revealed the absence of prezygotic phenological isolation in both contact zones. Overall, the negative effect of intraspecific pollination on fruit set indicated the compatibility of hybridization between the two oak species. However, the interaction between population and pollination treatment indicated that the fruit set after intraspecific pollen was significantly more than that after interspecific pollen in population NA but not in population Dlw, which suggested that some mechanisms during seed maturation were responsible for aborting the hybrid embryos in the ancient but not recent hybridization zone. Moreover, the proportion of hybrid seeds produced by both species in the ancient zone was significantly lower than that in the recent zone of secondary contact. The proportion of hybrid seeds simulated to form, assuming both random mating and an absence of postpollination barriers, was significantly higher than the proportion actually detected in the ancient zone, but not in the recent zone. These results suggest significantly stronger early-acting postzygotic isolation between the two oak species in the ancient relative to the recent secondary contact zone.

Prezygotic barriers are usually thought to contribute more to total reproductive isolation than postzygotic barriers in natural populations because prezygotic barriers reduce interspecific pollen transfer [17, 20]. In secondary contact zones, reinforcement selection suggests that traits capable of increasing prezygotic isolation could evolve and be reinforced to select against unfit hybrids [15, 35]. However, a recent review has speculated that prezygotic barriers could be more easily eroded under global change than postzygotic barriers [23]. When closely related species come into contact, geographical and phenological barriers may be eroded, which can lead to a degradation of prezygotic isolation in secondary contact zones [23, 36, 37]. In our study, we found no evidence for the phenological isolation because flowering time overlapped between the two oak species in both contact zones. Moreover, interspecific pollinations were compatible in both *Quercus* species because the full statistics model indicated that the fruit set after interspecific pollination was not less than that after intraspecific pollination (Fig. 1, Additional file 1: Table S1). When hybridization was fully compatible, it is not unusual to expect that intraspecific pollination may decrease the fruit set because the intraspecific pollen donors were more closely related to the pollen receptors than interspecific pollen donors.

Compared with prezygotic barriers, postzygotic barriers would be expected to be stronger in the ancient contact zone of two oak species, if postzygotic isolation are more intense between more diverged lineages [26, 27]. Based on the differences in genetic divergence and co-occurrence history between *Q. mongolica* and *Q. liaotungensis* [31, 34, 38], we expected to observe stronger postzygotic barriers in the ancient contact zone than in the recent contact zone. Because interspecific fertilization was compatible in both species, the abortion of hybrid embryos may lead to a reduced hybrid seed set [17]. Our results from hand pollination and natural hybridization were consistent with these expectations. First, the interaction between population and pollination treatment indicated that the fruit set after intraspecific pollination was significantly more than that after interspecific pollination in population NA but not in population Dlw, whenever *Q. mongolica* or *Q. liaotungensis* comprised the maternal species (Fig. 1, Additional file 1: Table S1). Second, the proportion of hybrid seeds under natural pollination in the ancient contact zone was much lower than that in the recent contact zone (Fig. 2). The proportion of hybrid seeds in the recent contact zone was 0.885 ± 0.036 for *Q. mongolica* and 0.682 ± 0.062 (2013) and 0.689 ± 0.044 (2014) for *Q. liaotungensis*, while the proportion was sharply reduced to less than 30% in the ancient contact zone (Fig. 2). Both the results suggested stronger early-acting postzygotic barriers in the ancient contact zone.

The relative abundance and spatial distribution of parent species and hybrid trees would influence the proportion of hybrid seeds in the same population [5]. To distinguish the effects of relative abundance on the production of hybrid seeds, we simulated the seed genotypes without any postpollination barriers using HYBRIDLAB and found that the simulated proportion of hybrid seeds was similar to the proportion obtained under natural conditions in the recent contact zone when *Q. mongolica* and *Q. liaotungensis* comprised the maternal plants (Fig. 2). However, the simulated proportion of hybrid seeds was much higher than the proportion under natural conditions in the ancient contact zone (Fig. 2). Therefore, the significantly lower proportion of hybrid seeds under natural conditions compared with simulated conditions in the ancient contact zone largely resulted from the abortion of fertilized embryos during the development of seeds. Collectively, these results are consistent with the expectation that stronger postzygotic barriers had a stronger inhibitory effect on hybridization in the ancient contact zone compared with the recent secondary contact zone because selection against hybrids had sufficient time to strengthen the postzygotic isolation between *Q. mongolica* and *Q. liaotungensis* in the ancient but not in the recent secondary contact zone.

Reduced hybrid viability and fertility often occur because of postzygotic barriers, even when hybrid seed matures, as detected in certain plants [39, 40]. For example, the F_1_ hybrids of *Trillium tschonoskii* as the maternal parent and *T. camschatcense* as the paternal parent failed to reach maturity [41], and the germination rates of hybrid seeds between *Quercus robur* and *Q. petraea* were reduced [42]. In the present study, we found that the probability of sampling a hybrid tree did not decrease with increasing DBH in both the ancient and recent secondary contact zones (Fig. 3). Our results suggest that hybrid trees did not show an increase in mortality during their growth and postzygotic barriers did not occur after germination if DBH was assumed to increase with the growth of the oak trees. The high frequency of hybrid trees in the contact zone of the two oak species has caused taxonomic confusion as indicated by the difficulty in distinguishing hybrid trees from parental oak trees [38].

## Conclusions

Our study provides the first comparison of reproductive isolation between ancient and recent secondary contact zones of two closely related oak species. The results demonstrate that postzygotic barriers during seed maturity account for the largest contribution to the total reproductive isolation between the two species, particularly in the ancient contact zone, thereby maintaining the species integrity. In the recent secondary contact zone, neither pre- nor postzygotic barriers were well developed, which led to a high frequency of natural hybridization and morphological and taxonomic confusion.

## Methods

### Study system

*Quercus liaotungensis* Koidz. and *Q. mongolica* Fisch. Ex Ledeb. (Fagaceae) are two closely related oak species of temperate broadleaved forests in China [31]. These oaks are monoecious and wind pollinated. Male catkins are solitary in leaf axils towards the base of branchlets or in paniculate clusters on lateral or subterminal shoots, and female flowers are scattered in leaf axils on the apical part of young shoots. The two species are sympatric in certain areas of North and Northeast China, although the range of *Q. liaotungensis* extends farther west into West China, and the range of *Q. mongolica* extends farther north into Russia and east into Korea, South Korea and Japan [31, 34]. Our study was conducted in populations NA (44°21′ N, 129°32′ E) and Dlw (39°58′ N, 115°26′ E) described in Zeng et al. [34]. A previous study suggested that the NA and Dlw populations are located in ancient and recent secondary hybridization zones, respectively. All the plant materials involved in this study were identified by Prof. Wan-Jin Liao and Yan-Fei Zeng and the voucher specimens were deposited in Beijing Normal University.

### Genotype scoring and individual assignment

We collected buds, seeds, and leaves to identify the species status of each individual because hybridization between the two oak species generated morphologically intermediate individuals and hence the two species cannot be straightforward distinguished from each other based on morphological traits [38]. All the materials were sampled from natural populations and no specific permission was needed to collect such samples. Genomic DNA was extracted from each bud, seed, or leaf using an N96 Plant Genomic DNA Kit (Tiangen, Beijing, China). Of the 19 nuclear SSR (simple sequence repeat) loci [31], we scored the following 7 loci with the highest power to distinguish the two oak species: ssrQpZAG36, ssrQpZAG15, ssrQpZAG110, quru-GA-0 M07, ssrQrZAG87, ssrQrZAG101, and ssrQrZAG112. The PCR amplification followed the procedures described in Zeng et al. [31]. The labeled PCR products were analyzed on an Applied Biosystems 3730 Genetic Analyzer with a LIZ500 size standard (Applied Biosystems, Grand Island, New York, USA) and the SSR genotyping was scored using GeneMapper v3.7 (Applied Biosystems, Grand Island, New York, USA).

Because no species-specific loci were detected [31], we used a Bayesian clustering method in STRUCTURE 2.3.4 [43] to identify each individual with *Q. mongolica*, *Q. liaotungensis*, or mixed ancestry. The datasets used in these assignment analyses included two parts: one was the 7-locus SSR data of individuals studied in Zeng et al. [34] (defined as preset data), and the other was the 7-locus SSR data of individuals we scored in the present study (defined as new data). Individuals from populations Dlw and NA were analyzed separately. In the analysis of individuals from population Dlw, the preset data included all individuals from the 38 populations studied in Zeng et al. [34]. In the analysis of individuals from population NA, the preset data included individuals from 28 populations. The 10 *Q. liaotungensis* populations from Zeng et al. [34] located in Northwest-North China were excluded because *Q. liaotungensis* populations from Northwest-North China and Northeast China are significantly genetically differentiated [34]. The program was run for K = 2 to detect the probability (*Q*) of each individual being assigned to the two clusters (two species: *Q. mongolica* and *Q. liaotungensis*) without prior population information. We assumed correlated allele frequencies and an admixed origin of populations. The burn-in was set to 50,000 and the run length was set to 200,000. Ten replicates were analyzed, and the results for the same data set were summarized by CLUMPP [44]. When the probability assigned to one cluster was greater than 0.9, we defined the individual belonging to that species; otherwise, the individual was a hybrid between the two species. The cut-off value was set to 0.85 only for the hand pollination experiments to obtain more parental plants.

### Flowering phenology

We recorded the flowering phenology in populations NA and Dlw to investigate whether differentiation in flowering phenology prevented hybridization between the two oak species. In late April of 2012 and 2013, we collected buds from 30 oak trees in population NA and 78 oak trees in population Dlw, respectively. Based on the assignment described above, we chose four *Q. liaotungensis* trees and three *Q. mongolica* trees in population NA in 2012 and recorded the pollen dispersal of each tree from 15th May to 25th May, and in population Dlw, we chose 16 *Q. liaotungensis* trees and seven *Q. mongolica* trees in 2013 to record the duration of pollen dispersal from the 10th to the 25th of May. We checked almost all flowers on each observed tree each day and recorded whether flowers dispersed pollen until the dispersal of pollen ceased for almost all flowers.

### Hand pollination experiments

We conducted hand pollination experiments to investigate whether interspecific pollination incompatibility prevented hybridization between the two oak species. Based on the assignment results, we chose six *Q. liaotungensis* trees and two *Q. mongolica* trees as maternal plants in population NA in 2012. From the six *Q. liaotungensis* trees, we randomly selected 281 female flowers from 42 lateral branches for interspecific hand pollination and 438 female flowers from 54 branches for intraspecific hand pollination. We removed all male catkins from the chosen terminal branches in the early development stage of the male catkins and bagged all female flowers approximately 5 days before the stigmas were receptive. We also bagged a number of male catkins and collected pollen grains for hand pollination. When female flowers were receptive, we brushed the collected pollen on the stigmas. To reduce the potential pollen contamination from the air, we used an unused, large plastic bag to rebag the chosen terminal branches, and we performed the hand pollination in this bag. The bags were removed 7 days after hand pollination. From the two *Q. mongolica* maternal trees, 23 female flowers from seven branches and 35 female flowers from 15 branches were hand pollinated with inter- and intraspecific pollen, respectively.

In population Dlw, we chose five *Q. liaotungensis* trees and 11 *Q. mongolica* trees as maternal plants for hand pollination in 2013. From the five *Q. liaotungensis* trees, we chose 418 female flowers from 31 branches and 209 female flowers from 22 branches for the inter- and intraspecific hand pollination, respectively. From the 11 *Q. mongolica* trees, we chose 304 female flowers from 33 branches and 321 female flowers from 39 branches for the inter- and intraspecific hand pollination, respectively. We counted the number of developing and aborted ovaries from each branch at 10 and 30 days after hand pollination and calculated the fruit set as the number of developing ovaries divided by the number of female flowers on each branch that we hand pollinated.

We evaluated the effects of hand pollination treatment, maternal species, and population on fruit set by fitting generalized linear mixed models (GLMMs) with a binomial distribution and logit link function using lme4 [45]. Within this model, the number of developing and aborted ovaries were responsible variables, the hand pollination treatment, maternal species, and population were set as the fixed factors with full interactions, and the maternal trees and measurement time were set as the random factors.

### Detecting hybrid seeds under open and simulated pollination conditions

To estimate the proportion of hybrid seeds under natural pollination, we collected seeds from individuals of pure species for SSR genotyping and species assignment. Based on the cut-off value of 0.9, we identified 17 *Q. liaotungensis* and 10 *Q. mongolica* trees in population NA in 2012, 4 *Q. liaotungensis* trees in population Dlw in 2013, and 5 *Q. liaotungensis* and 4 *Q. mongolica* trees in population Dlw in 2014. We attempted to collect more than 30 fruits from each of the chosen pure oak trees, and we extracted the genomic DNA from each embryo for the subsequent SSR genotyping. Finally, we identified each seed as an individual of *Q. liaotungensis* or *Q. mongolica* or as a hybrid according to the SSR genotype assignment analysis described above with a cut-off value of 0.9. The seed was assumed to originate from intraspecific pollination when both the seed and the maternal oak tree were assigned to the same species, and was considered a pure seed; otherwise, the seed was considered a hybrid seed. The proportion of hybrid seeds was calculated as the number of seeds fertilized by non-intraspecific pollen divided by the number of seeds scored for each tree. In total, 785 seeds were scored from population NA and 253 seeds were scored from population Dlw.

To investigate the effects of maternal species and population on the proportion of hybrid seeds under natural pollination, we fitted generalized linear mixed models (GLMMs) with a binomial distribution and logit link function. Within this model, the number of hybrid seeds and pure seeds were responsible variables, the maternal species and population were set as the fixed factors with interaction, and the year was the random factor.

To evaluate the expected proportion of hybrid seeds without any postpollination barriers in each population, we used HYBRIDLAB [46] to generate seed genotypes for each sampled mother tree. For each population, the seed genotypes were simulated separately for each mother tree as follows. The mother trees that we sampled to detect the hybrid seeds under natural pollination were set as parental 1, and all other trees with DBH > 5 cm (described in the following “Hybrid growth costs” section) were assumed to have the ability to fertilize and thus were set as parental 2. A total of 30 genotypes of the F_1_ offspring were generated by simulating random mating between parental 1 and 2. The same assignment analyses were applied to each of the simulated offspring to identify their species status, and the proportion of simulated hybrid seeds was calculated under random mating for each mother tree. We used paired t-tests to test whether the real proportion of hybrid seeds under natural pollination differed from the simulated proportion of hybrid seeds under random mating.

### Hybrid growth costs

To evaluate whether the hybrids had an increased growth disadvantage, we sampled the oak trees in populations NA and Dlw. We assumed that the tree diameter will increase with age, and we measured the DBH as an index of tree age. In each population, we adopted a grid-sampling method and collected samples every five meters from both large and small trees. We collected one leaf from each tree and sampled 243 trees in population NA and 341 trees in population Dlw. All the sampled individuals of each population were subjected to SSR genotype scoring and assignment analyses to identify the species status. We expected that the proportion of individual trees that were hybrids would decrease with tree age if hybrid offspring suffered serious disadvantages during their growth.

To evaluate whether growth costs increased with hybrid growth, we first used a two-sample Kolmogorov-Smirnov test to determine whether the DBHs of the hybrids and parental species individuals were drawn from the same distribution. Then, we tested whether the probability of randomly sampling a hybrid tree from a population would decrease with increasing DBH. Based on the assignment analyses of the randomly sampled trees in each population, we valued the hybrid individuals as 1 and the individuals representing the parental species as 0, and modeled these 1/0 data by fitting a logistic regression with DBH as the explanatory variable. All statistical analyses were conducted using R [47].

## Additional files

Additional file 1: **Table S1.** The generalized linear mixed models for fruit set. We simplified the full model by dropping the insignificant variables or interaction until no more could be dropped. We compared full model with simplified models by analysis of deviance. NA: population NA, intra: intraspecific pollination, QM: *Quercus mongolica*. (DOCX 17 kb)

Additional file 2: **Table S2.** The generalized linear mixed model for proportion of hybrid seeds. NA: population NA, QM: *Quercus mongolica*. (DOCX 15 kb)

## Abbreviation

DBH: Diameter at breast height
