# Supplementary material for: A comparison of reproductive isolation between two closely related oak species in zones of recent and ancient secondary contact
Source: BMC Evol Biol. 2019 Mar 6;19:70. doi: 10.1186/s12862-019-1399-y (PMC6404273; doi:10.1186/s12862-019-1399-y)
Supplement: Supplementary file 1 — Table S1. The generalized linear mixed models for fruit set. We simplified the full model by dropping the insignificant variables or interaction until no more could be dropped. We compared full model with simplified models by analysis of deviance. NA: population NA, intra: intraspecific pollination, QM: Quercus mongolica. (DOCX 17 kb) [file 12862_2019_1399_MOESM1_ESM.docx]

Table S1 The generalized linear mixed models for fruit set. We simplified the full model by dropping the insignificant variables or interaction until no more could be dropped. We compared full model with simplified models by analysis of deviance. NA: population NA, intra: intraspecific pollination, QM: *Quercus mongolica*.

| Model | Parameter | Estimate (mean ± se) | *P*-value | Model comparison | |
| --- | --- | --- | --- | --- | --- |
|  |  |  |  | Deviance | P-value |
| *Q. liaotungensis* and *Q. mongolica* | |  |  |  |  |
| Population*Treatment*Species | |  |  |  |  |
|  | Intercept | 0.992±0.839 | 0.237 |  |  |
|  | Population NA | 0.212±0.608 | 0.727 |  |  |
|  | Treatment intra | **-0.614±0.156** | **<0.001** |  |  |
|  | Species QM | **-1.953±0.596** | **0.001** |  |  |
|  | Population NA : Treatment intra | **0.651±0.217** | **0.003** |  |  |
|  | Population NA : Species QM | 0.956±1.090 | 0.380 |  |  |
|  | Treatment intra : Species QM | **1.004±0.218** | **<0.001** |  |  |
|  | Population NA : Treatment intra : Species QM | -0.039±0.618 | 0.950 |  |  |
| Population*Treatment+Treatment*species | |  |  | 0.826 | 0.662 |
|  | Intercept | 0.832±0.834 | 0.318 |  |  |
|  | Population NA | 0.536±0.507 | 0.291 |  |  |
|  | Treatment intra | **-0.619±0.151** | **<0.001** |  |  |
|  | Species QM | **-1.688±0.530** | **0.001** |  |  |
|  | Population NA : Treatment intra | **0.654±0.203** | **0.001** |  |  |
|  | Treatment intra : Species QM | **1.008±0.204** | **<0.001** |  |  |
|  |  |  |  |  |  |
| *Q. liaotungensis* | |  |  |  |  |
| Population*Treatment | |  |  |  |  |
|  | Intercept | -0.304±0.750 | 0.685 |  |  |
|  | Population NA | 0.266±0.491 | 0.578 |  |  |
|  | Treatment intra | **-0.613±0.156** | **<0.001** |  |  |
|  | Population NA : Treatment intra | **0.655±0.216** | **0.002** |  |  |
|  |  |  |  |  |  |
| *Q. mongolica* | |  |  |  |  |
| Population*Treatment | |  |  |  |  |
|  | Intercept | **1.382±0.520** | **0.008** |  |  |
|  | Population NA | 0.929±0.952 | 0.329 |  |  |
|  | Treatment intra | **0.389±0.154** | **0.012** |  |  |
|  | Population NA : Treatment intra | 0.623±0.579 | 0.282 |  |  |
| Treatment | |  |  | 3.307 | 0.191 |
|  | Intercept | **1.629±0.529** | **0.002** |  |  |
|  | Treatment intra | **0.444±0.149** | **0.003** |  |  |
