# Supplementary material for: A comparison of reproductive isolation between two closely related oak species in zones of recent and ancient secondary contact
Source: BMC Evol Biol. 2019 Mar 6;19:70. doi: 10.1186/s12862-019-1399-y (PMC6404273; doi:10.1186/s12862-019-1399-y)
Supplement: Supplementary file 2 — Table S2. The generalized linear mixed model for proportion of hybrid seeds. NA: population NA, QM: Quercus mongolica. (DOCX 15 kb) [file 12862_2019_1399_MOESM2_ESM.docx]

Table S2 The generalized linear mixed model for proportion of hybrid seeds. NA: population NA, QM: *Quercus mongolica*.

| Model | Parameter | Estimate (mean ± se) | *P*-value |
| --- | --- | --- | --- |
| Population*Species |  |  |  |
|  | Intercept | **0.793±0.160** | **<0.001** |
|  | Population NA | **-1.823±0.189** | **<0.001** |
|  | Species QM | **1.772±0.491** | **<0.001** |
|  | Population NA : Species QM | **-1.710±0.518** | **0.001** |
